# Acupuncture in chronic aspecific low back pain: a Bayesian network meta-analysis

**DOI:** 10.1186/s13018-022-03212-3

**Published:** 2022-06-20

**Authors:** Alice Baroncini, Nicola Maffulli, Jörg Eschweiler, Friedrich Molsberger, Alexandra Klimuch, Filippo Migliorini

**Affiliations:** 1 Department of Orthopaedics, Trauma, and Reconstructive Surgery, RWTH Aachen University Hospital, 52074 Aachen, Germany; 2 Department of Medicine, Surgery and Dentistry, University of Salerno, 84081 Baronissi, SA Italy; 3 Faculty of Medicine, School of Pharmacy and Bioengineering, Keele University, Stoke-on-Trent, ST4 7QB England; 4 Centre for Sports and Exercise Medicine, Barts and the London School of Medicine and Dentistry, Queen Mary University of London, London, E1 4DG England; 5 Acupuncture Study Group, Grafing, Germany

**Keywords:** Acupuncture, Low back pain, Verum acupuncture, Individualized acupuncture, Sham acupuncture

## Abstract

**Background:**

This Bayesian network meta-analysis investigated the available randomized control trials (RCTs) to point out which acupuncture protocol is the most effective for chronic aspecific low back pain (LBP). Efficacy was measured in terms of pain (Visual Analogic Scale, VAS) and disability (Roland Morris Disability Questionnaire, RMQ), Transcutaneous Electrical Nerve Stimulation (TENS).

**Methods:**

PubMed, Google scholar, Embase, and Scopus were accessed in March 2022. All the RCTs comparing two or more acupuncture modalities for aspecific chronic LBP were accessed. Only studies which investigated the efficacy of acupuncture on patients with symptoms lasting a minimum of 1.5 months, or with at least three episodes in the previous 12 months, were considered eligible. The Review Manager Software (The Nordic Cochrane Collaboration, Copenhagen) was used for the methodological quality assessment. The STATA Software/MP, Version 14.1 (StataCorporation, College Station, Texas, USA), was used for the statistical analyses. The NMA was performed through the STATA routine for Bayesian hierarchical random-effects model analysis.

**Results:**

Data from 44 RCTs (8338 procedures) were retrieved. 56% of patients were women. The mean age of the patients was 48 ± 10.6 years. The mean BMI was 26.3 ± 2.2 kg/m^2^. The individual group (95% confidence interval (CI) 2.02, 7.98) and the standard combined with TENS (95% CI 2.03, 7.97) demonstrated the highest improvement of the RMQ. The VAS score was lower in the standard combined with TENS group (95% CI 3.28, 4.56). Considering the standard acupuncture group, different studies used similar protocols and acupuncture points and the results could thus be compared. The equation for global linearity did not find any statistically significant inconsistency in any of the network comparison.

**Conclusion:**

Verum acupuncture is more effective than sham treatment for the non-pharmacological management of LBP. Among the verum protocols, individualized acupuncture and standard acupuncture with TENS were the protocols that resulted in the highest improvement in pain and quality of life.

***Level of Evidence*:**

Level I, Bayesian network meta-analysis of RCTs.

## Introduction

Low back pain (LBP) is common, leading to relevant economic burden [1, 2]. On average, the lifetime prevalence of LBP is as high as 80% [3, 4]. LBP is aspecific in most cases, and no pathoanatomical cause can be found [5]. In such patients, management aims to reduce symptoms and disability, allowing the return to daily life activities and participation in physiotherapy [5]. Current guidelines recommend physiotherapy as a first-line treatment for chronic LBP [6]. As some patients do not experience sufficient benefit from physiotherapy alone, further options are required. Pharmacotherapy is the second step in the management of chronic aspecific LBP [6]. NSAIDs and opiates represent the most successful treatment options [7, 8]. However, potential side-effects of pharmacotherapy, along with the risk of opioids addiction, make this option viable and safe only in the short term. As the number of patients with chronic aspecific LBP increases, so does the demand for safe and effective therapies. Among these, acupuncture has been widely investigated in recent times as possible options, proving to be a safe and effective therapy for chronic aspecific LBP [9–11]. Many different acupuncture protocols have been proposed for the management of chronic aspecific LBP. This Bayesian network meta-analysis investigated the available randomized control trials (RCTs) to try and identify which acupuncture protocol is the most effective for chronic aspecific LBP. Efficacy was measured in terms of pain (Visual Analogic Scale, VAS) and disability (Roland Morris Disability Questionnaire, RMQ) to investigate the efficacy of acupuncture on different aspects of the patients’ overall quality of life.

## Material and methods

### Search strategy

This Bayesian network meta-analysis was conducted according to the PRISMA extension statement for reporting of systematic reviews incorporating network meta-analyses of healthcare interventions [12]. A guide protocol was preliminary drafted:

- P (population): Chronic low back pain;
- I (intervention): Acupuncture;
- C (comparison): Standardized, sham, individual, auricular, electroacupuncture, acupuncture combined with Transcutaneous Electrical Nerve Stimulation (TENS);
- O (outcomes): VAS, RMQ.

### Data source and extraction

Two authors (A.M. and A.B.) independently performed the literature search in April 2022. The following databases were accessed: PubMed, Google scholar, Embase, and Scopus. The following keywords were used in combination: *low, lumbar, back, lower, spine, pain, disability, management, therapy, treatments, acupuncture, auricular, tens, electroacupuncture, auricular, individualized, standardized, visual analogic scale, vas, Roland Morris questionnaire*. The same authors independently performed the initial screening. If the title and abstract matched the topic, the article full-text was accessed. A cross reference of the bibliographies was also performed. Disagreement was debated and solved by a third author (N.M.).

### Eligibility criteria

All the RCTs comparing two or more acupuncture modalities for aspecific chronic LBP were accessed. Only studies which investigated the efficacy of acupuncture on patients with symptoms lasting a minimum of 1.5 months, or with at least three episodes in the previous 12 months were considered eligible. Only studies involving patients aged 18 to 75 were considered. Aspecific LBP was defined as pain that was not arising from a specific pathoanatomical condition [5]. Given the authors language capabilities, articles in English, German, Italian, French and Spanish were eligible. Only level I of evidence studies, according to Oxford Centre of Evidence-Based Medicine [13], were considered. Studies reporting data over acupressure or percutaneous electrical nerve stimulation were not considered. Studies reporting the efficacy of acupuncture in patients with acute pain were not included, nor were those investigating the cervicothoracic segments. Studies investigating patients with neurological deficits were excluded, along with studies including patients with radicular pain (unless the radicular pain was only a minor complaint). Editorials, reviews, letters, opinion, technical notes, comments were not eligible, nor were biomechanical, cadaveric, or animal studies. Only articles reporting quantitative data under the outcomes of interest were considered for inclusion. Missing data under the outcomes of interest warranted exclusion from this study.

### Data extraction

Two authors (A.M. and A.B.) independently performed data extraction the resulting articles for inclusion. Study generalities (author, year, journal, design, length of the follow-up) and patient demographic at baseline were extracted (number of samples and related mean BMI and age, percentage of female). For every treatment, the following data were retrieved: VAS, RMQ, adverse events. The groups considered were standard acupuncture alone, standard acupuncture with TENS, electroacupuncture, individualized acupuncture, and auricular acupuncture. The treatment group was classified as individualized acupuncture when the treating physician chose the needling points for each patient, without following a specific protocol. The treatment was defined as standard acupuncture when all patients were treated with the same protocol. Sham acupuncture was defined as the needling of points other than the known acupuncture points: as shallow needling of acupuncture points may also elicit a fibroblast reaction, this method was considered as a form of verum acupuncture [14]. The association of acupuncture with electrostimulation was considered separately.

### Risk of bias assessment

The risk of bias assessment was conducted by two authors (A.M. and A.B.) independently. The Review Manager Software (The Nordic Cochrane Collaboration, Copenhagen) was used for the methodological quality assessment. The following risk of bias were evaluated: selection, detection, reporting, attrition, and other source of bias. For each comparison, the risk of bias was investigated using funnel plots. Plot asymmetries relates to greater risk of bias.

### Statistical analysis

The statistical analyses were conducted by the senior author (F.M.). Baseline comparability was assessed through the IBM SPSS software. The analysis of variance (ANOVA) was used for analysis, with *P* values > 0.1 considered satisfactory. All other treatments rather than standardized, individual, auricular, electroacupuncture, combined with TENS, sham acupuncture were included in the “control group” and excluded from the analysis. The STATA Software/MP, Version 14.1 (StataCorporation, College Station, Texas, USA) was used for the statistical analyses. The NMA was performed through the STATA routine for Bayesian hierarchical random-effects model analysis. The inverse variance method was used for analysis, with standardized mean difference (STD) effect measure. The overall inconsistency was evaluated through the equation for global linearity via the Wald test. If the *P* value < 0.5, the null hypothesis cannot be rejected, and the consistency assumption could be accepted at the overall level of each treatment. Both confidence (CI) and percentile (PrI) intervals were set at 95%. For each comparison, edge plots were performed to display amount and interpolations of direct comparisons; interval plots were performed to rank the treatments according to their effect measure; and asymmetry of the funnel plots was associated with the greater risk of publication bias.

## Results

### Search result

**Fig. 1:**
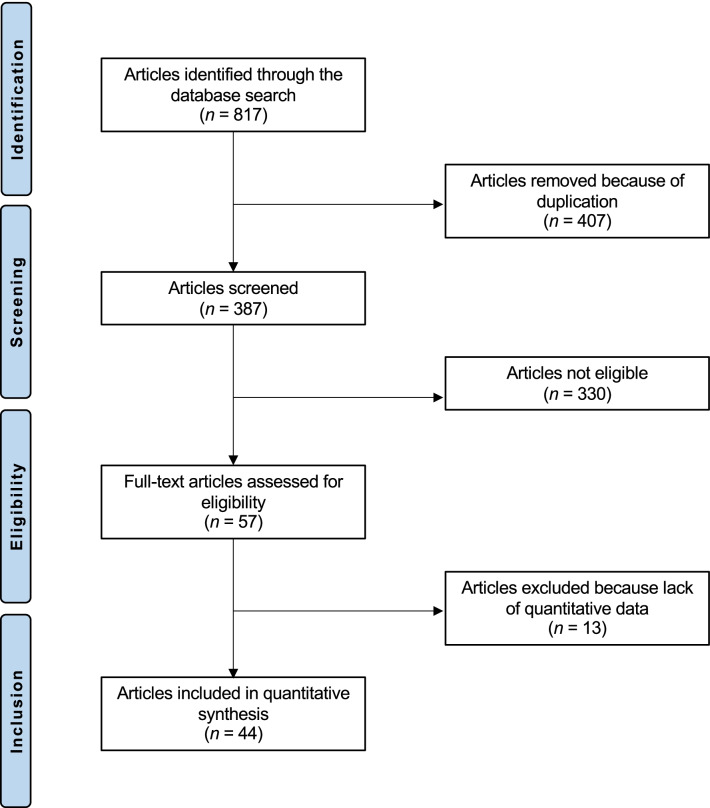
Flowchart of the literature search

The literature search resulted in 817 RCTs. Four hundred and seven were excluded as they were duplicates. A further 330 studies were incompatible with the eligibility criteria: type of study (*N* = 89), not matching the topic of interest (*N* = 193), acupressure or percutaneous electrical nerve stimulation (*N* = 15), acute LBP (*N* = 11), concerning cervicothoracic segments (*N* = 5), presence of severe neurologic impairment or spine deformities (*N* = 3), acute setting (*N* = 2), old age (*N* = 1), language limitations (*N* = 2), other (*N* = 9). Another 13 studies were excluded as they did not report quantitative data under the outcomes of interest. This left 44 RCTs for the present study. The literature search results are shown in Fig. 1.

### Methodological quality assessment

**Fig. 2:**
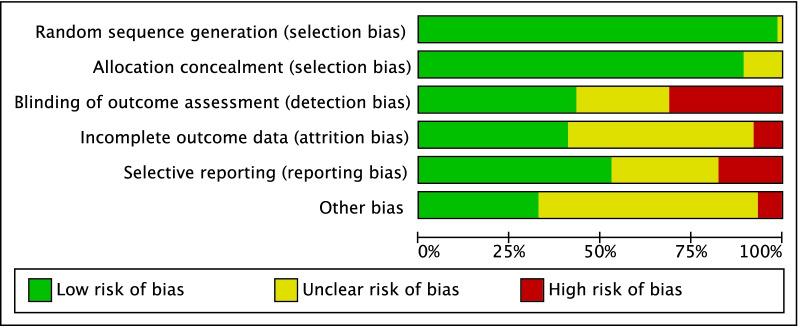
Methodological quality assessment

Given the randomized design of the included studies, the risk of selection bias was low. Assessor blinding was performed in 75% (33 of 44) studies; however, the blinding methods were often biased, and the overall risk of detection bias was moderate. The risk of attrition and reporting biases were low to moderate, as was the risk of other bias. Concluding, the methodological quality assessment showed low to moderate risk of bias (Fig. 2).

### Patient demographics

Data from 8338 procedures were retrieved. 56% of patients were women. The mean age of the patients was 48 ± 10.6 years. The mean BMI was 26.3 ± 2.2 kg/m^2^. The ANOVA test found moderate baseline comparability in symptoms duration (*P* > 0.05). Patient demographics are shown in Table 1.

**Table 1:** Generalities and patient baseline of the included studies

Author, year	Journal	Treatment	Type of protocol	Patients (*n*)	Follow-up (months)	Mean age	Women (%)	Mean BMI
Brinkhaus et al. [15]	*Arch Intern Med*	Individualized	12 sessions of 30 min over 8 weeks (2 sessions in each of the first 4 weeks, followed by 1 session per week in the remaining 4 weeks)	147	13	59.1	64	26.7
		Sham		75		58.2	75	26.2
		Control	Patients in the waiting list group did not receive acupuncture treatment for 8 weeks after randomization	79		58.9	68	26.9
Camilotti et al. [16]	*Fisioter. Mov*	Physiotherapy/ Aquatic Therapy	Twice a week	15		61.7		27.8
		Auricular/scalp/standardized		15		57.3		27.6
		Control		14		61.2		28.7
Ceccherelli et al. [17]	*The Clinical Journal of Pain*	Standardized	8 sessions for 20 min; the first four sessions were carried out in 2 weeks, whereas the remaining four sessions were carried out once per week	21	3	41.7	24	
		Individualized		21		41.6	33	
Cherkin et al. [18]	*Arch Intern Med*	Individualized	Up to 10 massages or acupuncture treatments over 10 weeks were permitted	94	12	45.3	52	
		Control		78		45.7	69	
		Control		90		43.8	56	
Cherkin et al. [19]	*Arch Intern Med*	Individualized	2 treatments weekly for 3 weeks; then once weekly for 4 weeks	157	13	47.0	68	
		Standardized		158		49.0	56	
		Sham		162		47.0	60	
		Usual care	Patients received no study-related care; just the care, if any, they and their physicians chose (mostly medications, primary care, and physical therapy visits)	161		46.0	64	
Cho et al. [20]	*Spine*	Individualized	12 acupuncture sessions (approximately 2 times a week for 6 weeks)	65	6	42.4	83	23.9
		Sham		65		41.8	86	24.2
Comachio et al. [21]	*Journal of Acupuncture and Meridian Studies*	Individualized	1-h sessions, twice a week for 6 weeks	33	3	49.0	70	26.9
		Electroacupuncture		33		46.0	58	26.0
Di Cesare et al. [22]	*Complementary Therapies in Medicine*	Control	1 injection per week for 4 weeks	29	3	52.5	55	
		Standardized		33		52.5	55	
Giles et al. [23]	*Journal of Manipulative and Physiological Therapeutics*	Individualized	15- to 20-min appointments with subsequent low-volt electrical stimulation applied to the needles, 6 treatments applied in a 3- to 4-week period, (mean 6 treatments)	20	0	46.5	65	
		Control	Pills given for the defined 3- to 4-week treatment period, (mean 2 prescriptions)	21		35.0	81	
		Control	15 to 20-min appointments, 6 treatments applied in a 3- to 4-week period, (mean 6 treatments)	36		42.5	47	
Giles et al. [24]	*Spine*	Standardized	20-min appointments, 2 treatments per week, maximum treatment duration of 9 weeks	36	12	37.5	44	25.8
		Control	Maximum treatment duration of 9 weeks	43		39.0	43	25.8
		Control	20-min appointments, 2 treatments per week, maximum treatment duration of 9 weeks	36		39.0	49	25.8
Grant et al. [25]	*Pain*	Individualized	2 sessions of manual acupuncture weekly for 4 weeks	30	3	75.0		
		TENS		27		72.0		
Haake et al. [26]	*Arch Intern Med*	Individualized	Ten 30-min sessions, generally 2 sessions per week, and 5 additional sessions if, after the tenth session, patients experienced a 10% to 50% reduction in pain intensity	387	6	49.6	57	26.9
		Sham		387		49.2	64	26.5
		Control		388		51.3	58	26.3
Hasegawa et al. [27]	*Acupunct Med*	Standardized	5 sessions during 28 days	40	0	47.0	63	
		Sham		40		43.9	65	
Hunter et al. [28]	*Clin J Pain*	Physiotherapy	Supervised group exercise session lasting for 1 h a week for 6 weeks	27	6	43.2	59	
		Auricular	Manual AA for the first 6 weeks of the trial before each exercise session	24		42.4	67	
Inoue et al. [29]	*Acupunct Med*	Individualized	Once	15		68.0	27	
		Sham		16		70.0	38	
Itoh et al. [30]	*Complementary Therapies in Clinical Practice*	Standardized	1 weekly treatment over 5 weeks	7	2.5		63	
		TENS		7				
		Acupuncture and TENS		7				
		Control	No specific treatment	7				
Itoh et al. [31]	*Acupunct Med*	Individualized	2 phases of 3 weeks, 12 weeks in total. Each patient received a total of six 30 min treatments, one per week	9	2.75	70.1	71	
		Individualized		9		71.9		
		Standard		9		73.8		
Itoh et al. [30]	*Complementary Therapies in Clinical Practice*	Standardized	5 treatments, once per week	8	2.5		63	
		TENS		8				
		Acupuncture and TENS		8				
		Control		8				
Kalauokalani et al. [32]	*Spine*	Control	Up to 10 treatments within 10 weeks		0			
		Individualized						
		Control						
		Control		66		45.0	70	
		Control		69		43.0	57	
Kennedy et al. [33]	*Complementary Therapies in Medicine*	Individualized	Once or twice a week; min 3 and max 12 treatments over 4–6 weeks	23	3	46.5	46	
		Sham		22		44.6	58	
Kerr et al. [34]	*Clin J Pain*	Standard	The treatment program consisted of 6 of these sessions over a 6-week period. This reflected the routine pattern of attendance for outpatient physiotherapy. Patients were given a leaflet regarding their low back pain that included standardized advice and exercises	26	1.5	42.6	50	
		Placebo-TENS		20		42.8	65	
Leibing et al. [35]	*Pain*	Standardized	20 sessions (each 30 min) of traditional and standardized acupuncture over 12 weeks, plus active physiotherapy. In the first 2 weeks of treatment, acupuncture was done five times a week, and in the next 10 weeks once a week. Plus physiotherapy 26 sessions (each 30 min) over 12 weeks	40	13	47.9	55	26.1
		Sham	20 sessions (each 30 min) of minimal acupuncture over 12 weeks, plus active physiotherapy 26 sessions (each 30 min) over 12 weeks	45		49.0	60	25.9
		Control	Active physiotherapy with no other treatment 26 sessions (each 30 min) over 12 weeks	46		47.5	59	26.9
Liu et al. [36]	*Clin Rehab*	Standardized	Usual care + assigned acupuncture intervention according to group allocation. 30-min treatment sessions were administered twice weekly	15	3	30.0	60	23.9
		Standardized		15		37.1	60	27.0
		Standardized		15		30.8	73	27.4
Luo et al. [37]	*Journal of traditional Chinese medicine*	Auricular	18 treatments were provided over 7 weeks	54	6	39.0	20	
		Standardized		50		36.0	24	
		Control		48		37.0	19	
Macdonald et al. [38]	*Annals of the Royal College of Surgeons of England*	Individualized	Over a short period of time once a week. The maximum number of treatments between the two assessments was arbitrarily defined as 10	8	0		75	
		Control		9			67	
Mendelson et al. [39]	*The American Journal of Medicine*	Standardized	4 weeks of treatment, twice weekly	36	1	54.5	47	
		Control		41		53.6	56	
Meng et al. [40]	*Rheumatology*	Standardized	In addition to standard therapy, subjects in this group received acupuncture treatments twice a week for 5 weeks, for a total of 10 sessions	31	2.25	72.0	58	
		Control	5 weeks	24		70.0	63	
Miyazaki et al. [14]	*Clin J Pain*	Standardized	A washout period of at least 4 weeks took place between phases 1 and 2	42		20.8		22.7
		Control		39		21.0		25.1
Molsberger et al. [41]	*Pain*	Sham	In addition to the daily conservative orthopedic therapy, all patients received 12 sham treatments, (30 min, 3/ week)	61	3	50.0	46	
		Standardized	In addition to the conventional conservative orthopedic therapy, 12 verum acupuncture treatments (30 min, 3/ week)	65		49.0	45	
		Control	These patients received the daily conventional conservative orthopedic treatment over 4 weeks	60		49.0	53	
Pach et al.[42]	*Evidence-Based Complementary and Alternative Medicine*	Standardized	2 treatment sessions per week had to be applied, with a maximum number of 10 to 15 sessions depending on the patient’s individual needs. The needle retention time was about 25 min	78	6.5	59.3	54	27.2
		Individualized		72		56.1	63	27.0
Rajfur et al. [43]	*Medical science monitor*	TENS	A series of 15 treatments, 5 times a week (Monday to Friday) for a period of 3 weeks	20	0	50.2	55	26.7
		Acupuncture and TENS		20		52.1	60	27.0
		Control		19		47.8	58	26.3
		Control		21		48.7	62	26.1
		Control		22		52.1	59	25.9
		Control	Motor improvement exercises were used for 3 weeks, 5 times a week, from Monday to Friday	21		49.8	62	26.1
Sator-Katzenschlager [44]	*Pain Medicine*	Electroacupuncture	The acupuncture needles with the P-Stim™ devices were withdrawn 48 h after insertion in all cases and the acupuncture treatment was performed once a week for 48 h at home, for a total study period of 6 wk	61	1.5	54.1	71	26.6
		Acupuncture				53.1		25.3
Shankar et al. [45]	*Indian Journal of Physiology and Pharmacology*	Electroacupuncture	10 treatments over 3 weeks	30	0	36.2	53	22.9
		Control	10 days of Valdecoxib and 3 weeks of physiotherapy	30		34.5	80	22.0
		Control		30		35.9	57	23.3
Szczurko et al. [46]	*PLoS ONE*	Acupuncture	Twice per week to receive 24 treatments over a period of 12 weeks	39	3	45.3	56	28.7
		Control	Participants randomized to the control group received an educational booklet (causes of back pain, prognosis, appropriate use of imaging studies and specialists, and exercises for promoting recovery and preventing recurrences)	36		48.0	44	27.7
Thomas et al. [47]	*BMJ*	Individualized	10 individualized treatment sessions over 3 months	159	24	42.0	62	
		Control	Patients in the usual care group received NHS treatment according to their general practitioner’s assessment of need	80		44.0	58	
Witt et al. [48]	*American J Epidemiology*	Individualized	Maximum of 15 acupuncture sessions over 3 months	1451	6	53.1	58	
		Control	Conventional therapy	1390		52.6	57	
Yeung et al. [49]	*Journal of Alternative and Complementary Medicine*	Control	An hourly session each week for 4 consecutive weeks + daily 15 months of home exercise	26	3	55.6	81	24.2
		Standardized	EA three times/ week, 4 weeks	26		50.4	85	25.5
Tsui et al. [50]	*The journal of alternative and complementary medicine*	Electroacupuncture	Treatment for 20 min on a total of 6 acupuncture points. Treatment was delivered twice per week for 4 weeks (a total of 8 sessions)	14	1	39.7	76	
		Electroacupuncture		14		39.1	71	
		Control		14		40.9	62	
Tsukayama et al. [51]	*Acupuncture in medicine*	Electroacupuncture	Twice a week for 2 weeks	10	0	47.0	89	22.5
		TENS		10		43.0	80	22.2
Weiß et al. [52]	*The journal of alternative and complementary medicine*	Standardized	21-day inpatient rehabilitation and acupuncture twice weekly	74	3	49.8	27	30.0
		Control	21-day inpatient rehabilitation	69		51.7	39	28.8
Yoo et al. [53]	*Journal of Korean Medicine Rehabilitation*	Standardized	Treatment within 5 days. A total of 2 treatments	15	0	37.9		22.3
		Sham		15		37.9		23.9
Yuan et al. [54]	*Complementary Therapies in Medicine*	Individualized	10 treatments; 2 times/week	15	12	43.5	40	27.0
			10 treatments; 5 times/week	15		43.9	40	27.3
Yun et al. [55]	*The Journal of Alternative and Complementary Medicine*	Control	18 treatments were provided over 7 weeks, every other day for 3 weeks and then twice weekly for 4 weeks	64	11	35.0	22	
		Individualized		60		34.0	27	
		Control		63		33.0	19	
Zaringhalam et al. [56]	*Chinese Medicine Journal*	Standardized	Twice a week for 5 weeks	20	2.5	54.2	0	32.5
		Control	daily	20		55.1		29.2
		Control	Twice a week for 5 weeks	20		54.2		30.3
		Control		20		54.3		31.0

### Outcomes of interest

**Fig. 3:**
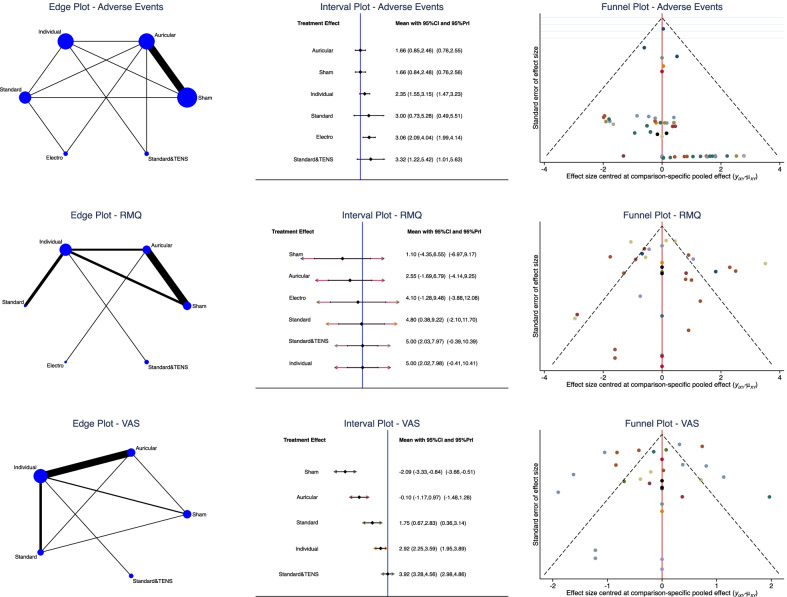
Results of the network comparisons

The individual group (SMD 5.00; 95% CI 2.02, 7.98) and the standard combined with TENS (SMD 5.00; 95% CI 2.03, 7.97) demonstrated the highest improvement of RMQ. The VAS score was lower in the standard combined with TENS group (SMD 3.92; 95% CI 3.28, 4.56). Considering the standard acupuncture group, different studies utilized similar protocols and acupuncture points and the results could thus be compared. The equation for global linearity did not find any statistically significant inconsistency in any of the network comparisons. Edge, interval, and funnel plots are shown in Fig. 3.

## Discussion

According to the main findings of the present Bayesian network meta-analysis, individualized acupuncture and the standard protocol with TENS may represent the most effective acupuncture strategies for the management of chronic aspecific LBP. These two treatment protocols showed the highest improvements of VAS and RMQ. As verum acupuncture scored better than sham treatment, the present study points to the efficacy of acupuncture in the management of aspecific chronic LBP. This finding further supports current guidelines which recommend acupuncture as one of the possible first-line, non-pharmacological management modality for aspecific chronic LBP [6].

These results are consistent with previous studies [10, 11], which observed a superiority of acupuncture compared to sham treatment [57, 58]. A meta-analysis by Amaral et al. observed only moderate-quality evidence in favor of acupuncture treatment for LBP; however, this study focused only on trigger point acupuncture in the geriatric population, and the results are not directly comparable with those of the present cohort [9]. Nascimiento et al. also observed poor outcomes for acupuncture for LBP in the geriatric population [59]. While no specific characteristics could be highlighted in patients who responded to acupuncture for chronic pain [60], the role of age on the effectiveness of the therapy in the setting of LBP deserves further investigation. A meta-analysis by Mu and colleagues found that acupuncture has only limited effect on pain relief and improvement of the quality of life in the immediate to short term [61]. Li et al. highlighted that a treatment duration of at least 5 weeks is required to achieve 80% of the maximum analgesic effect [62] of acupuncture. Thus, the relatively short timeframe considered by Mu et al. (one to 12 weeks) [61], along with the high risk of bias of the included studies [61], may explain the differences in the observed results. The literature regarding the use of acupuncture in the acute setting is limited and, so far, no studies have been directed to analyze the literature regarding different types of acupuncture for the treatment of acute LBP. Thus, a direct comparison of the presented results with those of the acute setting is not possible. Overall, acupuncture seems to be modestly effective for the management of acute LBP, and the available studies agree that more high-quality work are required to gain more evidence around this topic [63, 64]. Considering the reluctance of many physicians to prescribe acupuncture in patients with chronic LBP, it is fundamental to highlight the efficacy of this treatment in comparison with sham acupuncture. However, the finding that individualized treatment is more effective than standard treatment suggests that a precise diagnosis of chronic LBP according to the criteria of traditional Chinese medicine and the choice of specific acupuncture points may be key for the efficacy of the treatment. This result confirms the clinical experience of the authors. The association of standard protocols with TENS yielded however results similar to those of individualized acupuncture.

This study has several limitations. The most important limitation is the heterogeneous protocols used. Given the randomized design of patient allocation, the risk of selection bias was low. However, some studies did not perform patients or personnel blinding, thus increasing the risk of detection and performance biases, respectively. General health measures were seldom reported, as were also information about the duration of previous symptoms, and type of pain (e.g., central sensitization). Previous conservative strategies were barely reported. Given the lack of quantitative data, the ethnicity of the patients was not analyzed separately. Given the heterogeneity of the protocols used, it was not possible to analyze the effects of the associations of acupuncture with other treatments such as pharmacological management and physio- and psychotherapy. Also, some acupuncture techniques such as moxibustion, cupping or scalp acupuncture were excluded from the study as the available data were not sufficient to grant inclusion. As a multimodal approach is often advisable to tackle the different organic and psychosocial aspects of chronic LBP [6, 65–68], further studies will be required to investigate the efficacy of different treatment associations and protocols. Furthermore, the available studies only allowed for a precise analysis of the effects of acupuncture in the population < 75 years old: targeted studies for this segment of the population will be required to investigate the efficacy of acupuncture in the elderly.

## Conclusion

Verum acupuncture is more efficient than sham treatment for the non-pharmacological management of LBP. Among verum protocols, individualized acupuncture and standard acupuncture with TENS were the protocols that allowed for the highest improvement in pain and quality of life. 

## Data Availability

The data underlying this article are available in the article and in its online supplementary material.

## Abbreviations

RCTs: Randomized control trials
LBP: Low back pain
VAS: Visual analogic scale
RMQ: Roland Morris disability questionnaire
PRISMA: Preferred reporting items for systematic reviews and meta-analyses
BMI: Body mass index
ANOVA: Analysis of variance
NMA: Network meta-analysis
STD: Standardized mean difference
CI: Confidence interval
PrI: Percentile interval
